# Hopes for Modifications That Are Yet to Come

**Published:** 1918-09-07

**Authors:** 


### The London Hospital Correspondence.

### HOPES FOR MODIFICATIONS THAT ARE YET TO COME.

The revival of the controversy over the system still in force at the London Hospital has led to the formulation of some statements which have no justification in the facts of to-day's training at the London Hospital. One was in effect that nurses with less than two years' training in the wards have been sent out private nursing to the public. The correspondence we have published lately demonstrates that these unpardonable irregularities occurred before the year 1900, and no such cases are officially declared to have happened since. Next we were appealed to by Miss McKinley on behalf of the probationer nurse and nurses generally, and the position was one which had to be gone thoroughly into and examined on its merits with all the available evidence before us. This entailed the publication of Miss McKinley's letter, and our article upon it dealing with the subject, published on page 415 of The Hospital for August 10, so that the fullest opportunity should be afforded to every member of the nursing profession to know the charges and, if there was any substance in the floating statements to which Miss McKinley attached her name, the former or present probationer, if she existed in fact, should have an opportunity to state her case. We pointed out in our issue of August 10 that it was a remarkable fact that, during the whole discussion which has taken place on the system of training nurses at the London Hospital, no complaint had been addressed to us by a probationer nurse.

Miss McKinley's letter has been widely circulated through The Hospital. No support has been forthcoming from any aggrieved nurse in this connection. We are glad to note that in these circumstances Miss Helena McKinley, in her second letter (see The Hospital, August 24, page 457), as an honourable woman has hastened to express her regret and to withdraw her letter of July 27 which appeared on page 397 of The Hospital for August 3. It is a serious matter to make grievous charges of the kind in the absence of incontestable evidence that they are true and relate to present conditions resting upon incontrovertible facts. We hope this truth may be admitted and enforced by nurses generally, and taken to heart on occasion, as Miss McKinley has taken them, to her credit, with promptness and dispatch.

There remain other important matters in the London Hospital system to be dealt with and determined, as the correspondence published in *The Times* and The Hospital has demonstrated. It is our hope and expectation that certain adjustments will speedily follow, and that unity for the nursing profession may soon become the reality all who have its true welfare at heart so earnestly desire.

